# Relationships among oncostatin M, insulin resistance, and chronic inflammation: a pilot study

**DOI:** 10.20945/2359-3997000000357

**Published:** 2021-04-05

Arch Endocrinol Metab. 2020;64(1):38-44

Where you read:

**Murat Akarsu** ^1^

https://orcid.org/0000-0002-2675-4252

**Mehmet Hurşitoğlu** ^2^

https://orcid.org/0000-0002-9062-118X

**Zeki Toprak** ^2^

https://orcid.org/0000-0002-7411-3628

**Şengül Aydin Yoldemir** ^1^

https://orcid.org/0000-0003-4236-1181

**Özgür Altun** ^1^

https://orcid.org/0000-0003-1810-7490

**Ilkim Deniz Toprak** ^3^

https://orcid.org/0000-0002-9320-1252

**Mustafa Özcan** ^1^

https://orcid.org/0000-0002-6045-1394

**Gülden Yürüyen** ^4^

https://orcid.org/0000-0002-7757-696X

**Bilal Uğurlukişi** ^5^

https://orcid.org/0000-0003-3954-7161

**Mahmut Genco Erdem** ^6^

https://orcid.org/0000-0002-7783-8130

**Kerem Kirna** ^7^

https://orcid.org/0000-0003-0753-5713

**Pinar Demir** ^1^

https://orcid.org/0000-0002-9542-3174

**Gazi Çapar** ^8^

https://orcid.org/0000-0002-9857-0962

**Yücel Arman** ^1^

https://orcid.org/0000-0002-9584-6644

**Tufan Tükek** ^8^

https://orcid.org/0000-0002-4237-1163

^1^ Okmeydani Training and Research Hospital, Department of Internal Medicine, Istanbul, Turkey

^2^ Dr. Sadi Konuk Training and Research Hospital, Department of Internal Medicine, Istanbul, Turkey

^3^ Gaziosmanpaşa Taksim Training and Research Hospital, Department of Internal Medicine, Istanbul, Turkey

^4^ Fatih Sultan Mehmet Training and Research Hospital, Department of Internal Medicine, Istanbul, Turkey

^5^ Şişli Etfal Training and Research Hospital, Department of Internal Medicine, Istanbul, Turkey

^6^ Istinye Üniversity, Medical Park Hospital Department of Internal Medicine, Istanbul

^7^ Haseki Training and Research Hospital, Department of Internal Medicine, Istanbul, Turkey

^8^ Istanbul University, Medical Faculty, Department of Internal Medicine, Istanbul, Turkey

Should read:

**Murat Akarsu** ^1^

https://orcid.org/0000-0002-2675-4252

**Mehmet Hurşitoğlu** ^2^

https://orcid.org/0000-0002-9062-118X

**Zeki Toprak** ^2^

https://orcid.org/0000-0002-7411-3628

**Şengül Aydin Yoldemir** ^1^

https://orcid.org/0000-0003-4236-1181

**Özgür Altun** ^1^

https://orcid.org/0000-0003-1810-7490

**Ilkim Deniz Toprak** ^3^

https://orcid.org/0000-0002-9320-1252

**Mustafa Özcan** ^1^

https://orcid.org/0000-0002-6045-1394

**Gülden Yürüyen** ^4^

https://orcid.org/0000-0002-7757-696X

**Bilal Uğurlukişi** ^5^

https://orcid.org/0000-0003-3954-7161

**Mustafa Genco Erdem** ^6^

https://orcid.org/0000-0002-7783-8130

**Kerem Kirna** ^7^

https://orcid.org/0000-0003-0753-5713

**Pinar Demir** ^1^

https://orcid.org/0000-0002-9542-3174

**Gazi Çapar** ^8^

https://orcid.org/0000-0002-9857-0962

**Yücel Arman** ^1^

https://orcid.org/0000-0002-9584-6644

**Tufan Tükek** ^8^

https://orcid.org/0000-0002-4237-1163

^1^ Okmeydani Training and Research Hospital, Department of Internal Medicine, Istanbul, Turkey

^2^ Dr. Sadi Konuk Training and Research Hospital, Department of Internal Medicine, Istanbul, Turkey

^3^ GaziosmanpasÇa Taksim Training and Research Hospital, Department of Internal Medicine, Istanbul, Turkey

^4^ Fatih Sultan Mehmet Training and Research Hospital, Department of Internal Medicine, Istanbul, Turkey

^5^ SÇisÇli Etfal Training and Research Hospital, Department of Internal Medicine, Istanbul, Turkey

^6^ Beykent University Medical Faculty, Department of Internal Medicine, Istanbul, Turkey

^7^ Haseki Training and Research Hospital, Department of Internal Medicine, Istanbul, Turkey

^8^ Istanbul University, Medical Faculty, Department of Internal Medicine, Istanbul, Turkey

